# Blockade of Apoptosis Signal-Regulating Kinase 1 Attenuates Matrix Metalloproteinase 9 Activity in Brain Endothelial Cells and the Subsequent Apoptosis in Neurons after Ischemic Injury

**DOI:** 10.3389/fncel.2016.00213

**Published:** 2016-09-02

**Authors:** So Y. Cheon, Kyoung J. Cho, So Y. Kim, Eun H. Kam, Jong E. Lee, Bon-Nyeo Koo

**Affiliations:** ^1^Department of Anesthesiology and Pain Medicine, Severance Hospital, Yonsei University College of Medicine, SeoulSouth Korea; ^2^Anesthesia and Pain Research Institute, Yonsei University College of Medicine, SeoulSouth Korea; ^3^Department of Anatomy, Yonsei University College of Medicine, SeoulSouth Korea

**Keywords:** apoptosis signal-regulating kinase 1, matrix metalloproteinase, transient focal cerebral ischemia, hypoxia, brain endothelial cells

## Abstract

Conditions of increased oxidative stress including cerebral ischemia can lead to blood–brain barrier dysfunction via matrix metalloproteinase (MMP). It is known that MMP-9 in particular is released from brain endothelial cells is involved in the neuronal cell death that occurs after cerebral ischemia. In the intracellular signaling network, apoptosis signal-regulating kinase 1 (ASK1) is the main activator of the oxidative stress that is part of the pathogenesis of cerebral ischemia. ASK1 also promotes apoptotic cell death and brain infarction after ischemia and is associated with vascular permeability and the formation of brain edema. However, the relationship between ASK1 and MMP-9 after cerebral ischemia remains unknown. Therefore, the aim of the present study was to determine whether blocking ASK1 would affect MMP-9 activity in the ischemic brain and cultured brain endothelial cells. Our results showed that ASK1 inhibition efficiently reduced MMP-9 activity *in vivo* and *in vitro*. In endothelial cell cultures, ASK1 inhibition upregulated phosphatidylinositol 3-kinase/Akt/nuclear factor erythroid 2 [NF-E2]-related factor 2/heme oxygenase-1 signals and downregulated cyclooxygenase-2 signals after hypoxia/reperfusion. Additionally, in neuronal cell cultures, cell death occurred when neurons were incubated with endothelial cell-conditioned medium (EC-CM) obtained from the hypoxia/reperfusion group. However, after incubation with EC-CM and following treatment with the ASK1 inhibitor NQDI-1, neuronal cell death was efficiently decreased. We conclude that suppressing ASK1 decreases MMP-9 activity in brain endothelial cells, and leads to decreased neuronal cell death after ischemic injury.

## Introduction

After cerebral ischemia, an imbalance of oxidants can lead to the overproduction and accumulation of ROS. Oxidative stress is a critical component in the pathogenesis of ischemic brain injury and the disruption of BBB, which subsequently causes brain edema and neuronal death (Rosenberg, 1995; Allen and Bayraktutan, 2009). MMPs are one of the main factors regulating BBB integrity and neuronal cell death (Rosenberg, 1995; Rosenberg et al., 1996; Romanic et al., 1998). Especially, it is known that enhanced MMP-9 expression is associated with neuronal cell damage (Lee and Lo, 2004), apoptosis (Copin et al., 2005), and vascular disintegration. In contrast, the inhibition of MMP-9 ameliorates brain edema and infarct volume following cerebral ischemia (Rosenberg, 1995; Gasche et al., 1999; Asahi et al., 2001; Wang and Tsirka, 2005; Kelly et al., 2008; Yu et al., 2008; Hu et al., 2009).

Apoptosis signal-regulating kinase 1 is one of the major molecules activated in the intracellular system immediately following ischemic insults (Matsuzawa and Ichijo, 2008; Cheon et al., 2013). ASK1 is activated by oxidative stress in the pathogenesis of CNS injuries and disease such as ischemic stroke, Huntington’s disease, and Alzheimer’s disease (Minn et al., 2008; Cho et al., 2013; Kawarazaki et al., 2014). A previous study demonstrated that the induction of ASK1 expression promotes apoptotic cell death after ischemia, while the silencing ASK1 by small interfering RNA (siRNA) ameliorates cerebral infarction in the brain (Kim et al., 2011). The inhibition of ASK1 also exerts protective effects against ischemia induced brain edema (Song et al., 2015). ASK1 modulates vascular endothelial growth factor and aquaporin-1, which is associated with water homeostasis (Song et al., 2015). Moreover, in microarray analyses, ASK1 silencing reduced the expression of brain endothelial cell-related genes such as *Cdh1*, *Icam1*, *Gjb3* and *Sele*, which are the genes for cadherin1, intercellular adhesion molecule 1, gap junction protein beta1, and endothelial cell selectin respectively (Song et al., 2015). Although, many studies have shown that ASK1 is linked to vascular injury and neuronal cell death after cerebral ischemia, it remains unclear whether ASK1 affects MMP-9 activation, which plays a crucial role in ischemic stroke.

Therefore, we conducted the present study to determine whether the inhibition of ASK1 would attenuate MMP-9 activation in ischemic tissue after middle cerebral artery (MCA) occlusion and in brain endothelial cells after hypoxia/reperfusion (H/R) injury. In addition, we investigated whether the inhibition of ASK1 in endothelial cells under H/R condition would affect the neuronal cell fate by modulating apoptosis-related markers and cell viability.

## Materials and Methods

### Animals

All animals were housed in the controlled animal facilities with a 12 h light/dark cycle at Yonsei University. Mice were housed with 1–5 mice in the cage with free access to food and water. Animal experiment was followed in the Guide to the Care and Use of Laboratory Animals approved by the Association for Assessment and Accreditation of Laboratory Animal Care and the National Institutes of Health guideline. Ethical Committee of the Yonsei University approved this study. Adult male C57BL/6 mice (Orient, Seongnam, GyeongGi-Do, Korea; aged 8–12 weeks) were used in this experiment. The group was divided into three groups; normal control group (control), ischemia/reperfusion (I/R) group, and I/R+si-ASK1 group.

### Transient Focal Cerebral Ischemia/Reperfusion

Isoflurane vaporizer was used for anesthesia of mice with 2.0% isoflurane in 30% oxygen and 70% nitrous oxide. Mice were placed on homeothermic blanket and maintained rectal temperature at 37 ± 0.5°C. The MCA was blocked by inserting 6-0 nylon suture into the left external carotid artery (ECA) to induce transient focal cerebral ischemia (tFCI) for 60 min. The blood flow was restored by removing the nylon suture. Mice were housed the cages and monitored during experiments. At 24 h after reperfusion, mice were anesthetized by intraperitoneal injection of Zoletil mixture (30 mg/kg; VirvacLaboratories, Carros, France), and performed cardiac perfusion with normal saline.

### The bEnd.3 Cell culture

Mouse brain endothelial cells (bEnd.3, ATCC, Manassas, VA, USA) were cultured with Dulbecco’s Modified Eagle’s Medium high glucose (DMEM, Hyclone^TM^, GE Healthcare Life Sciences, Logan, UT, USA) adding fetal bovine serum (10%; FBS, GE Healthcare Life Sciences) and penicillin-streptomycin solution (1%; Thermo Scientific, Waltham, MA, USA). The endothelial cells were incubated in the CO_2_ chamber.

### The Neuro-2A Cell culture

Murine neuronal cell line, Neuro-2A, was cultured in DMEM high glucose cultured media, supplemented with 10% FBS (GE Healthcare Life Sciences) and 1% penicillin-streptomycin solution (Thermo Scientific). The neuronal cells were incubated at 37°C in a humid atmosphere in the presence of 5% CO_2_. To examine effects of endothelial cell-released factor on neuronal cells, neuronal cells were incubated with EC-CM which were collected after H/R injury.

### Hypoxia/Reperfusion Injury

Before OGD, the culture medium was discarded, washed with phosphate buffer saline (PBS) and changed with deoxygenated glucose-free balanced salt solution (BSS) under the anaerobic chamber (Forma Scientific, Inc., Marietta, GA, USA). Endothelial cells were exposed to OGD condition for 6 h. After hypoxia, BSS solution was discarded and changed into DMEM cultured media and cells were transferred to a CO_2_ incubator for 24 h. After reperfusion, cells and EC-CM were collected and stored at -80°C. NQDI-1 (600 nM, Tocris Bioscience, Bristol, UK), an inhibitor of ASK1, was treated in cultured media from 1 h before hypoxia to 6 h during hypoxia.

### siRNA Targeting ASK1

ASK1 gene was silenced by using siRNA (Ambion, Austin, TX, USA, sense, GCUCGUAAUUUAUACACUGtt; antisense, CAGUGUAUAAAUUACGAGCtt; 5 μM). A 100 μl solution of siPORT*Neo*FX (Ambion) and siRNA for ASK1 was injected into the lateral ventricle of mice (mediolateral 1.0 mm; anteroposterior 0.2 mm; dorsoventral 3.1 mm) with an osmotic pump (Alzet, Cupertino, CA, USA) for 3 days before ischemic injury.

### Cresyl Violet Staining

At 24 h after reperfusion, brain of mice was isolated after perfusion with saline. Each brain was fixed with 3.7% formaldehyde and stored at -80°C. Samples were sectioned coronally by a cryotome. Thickness of sections was 20 μm. Sections were stained with cresyl violet (Sigma-Aldrich, St. Louis, MO, USA) for 3 min and washed with distilled water for three times. Samples were mounted by using mounting medium (Vector Laboratories, Inc., Burlingame, CA, USA) and observed under microscope (Olympus, Tokyo, Japan).

### Double Staining for NeuN and TUNEL

Frozen brain sections were sliced by a microtome with 20 μm thickness. Sections were onto coating slide glass (MUTO Pure Chemicals Co., Ltd, Tokyo, Japan) and dried at room temperature. Frozen mouse brain coronal sections were washed with PBS for one times, and dipped into -20°C ethyl alcohol for 20 min, followed by 0.3% Triton X-100 diluted in PBS for 1 h. After washing with PBS, samples were blocked in 5% bovine serum albumin (BSA) diluted in PBS for 1 h and incubated with rabbit anti-NeuN antibody (Abcam, Cambridge, UK) at 4°C overnight. After washing with PBS, terminal deoxynucleotidyl transferase dUTP nick end labeling (TUNEL) assay for measurement for DNA fragmentation was performed followed by manufacture’s instruction (RocheDiagnostics, Indianapolis, IN, USA). Hoechst 33342 (ThermoFisher Scientific) or Propidium iodide (PI; Sigma-Aldrich) was used for counterstaining. After washing with PBS, stained brain were mounted with Vectashield (Vector Laboratories, Inc.). After then, sections were observed by using an LSM 700 confocal laser scanning microscope (Carl Zeiss, Thornwood, NY, USA).

### Western Blot Analysis

The tissues were lysate with a RIPA buffer (Biosesang, Inc., Seongnam, South Korea) with Halt^TM^ protease & Phosphatase inhibitor cocktail (1:100, Thermo Scientific). After centrifuged at 13000 rpm at 4°C for 20 min, the pellet was collected and protein concentration was measured by using Pierce^®^ BCA protein assay kit (Thermo Scientific). Sample buffer was added and boiled at 95°C for 5 min. Samples were transferred to polyvinylidene difluoride membranes (PVDF, Merck Millipore, Bedford, MA, USA). Membranes were blocked with 5% BSA and incubated in the rabbit anti-phosphatidylinositol 3-kinase (Santa Cruz Biotechnology, Santa Cruz, CA, USA), rabbit anti-pAkt (1:1000, Cell signaling, Danvers, MA, USA), rabbit anti-Akt (1:1000, Cell signaling), rabbit anti-nuclear factor erythroid 2 [NF-E2]-related factor 2 (1:1000, Santa Cruz Biotechnology), rabbit anti- HO-1 (1:1000, Abcam, Cambridge, UK), rabbit anti-Bcl-2 (1:1000, Abcam), mouse anti-Bax (1:1000, Merck Millipore), goat anti-cyclooxygenase-2 (1:500, Santa Cruz Biotechnology), rabbit anti-cleaved caspase-3 (1:1000, Santa Cruz Biotechnology), and rabbit anti-caspase-3 (1:1000, Merck Millipore) which is diluted in 2% BSA. Horseradish peroxidase-conjugated anti-rabbit, anti-goat, or anti-mouse IgG reagents (1:5000) were used as secondary antibodies (Jackson ImmunoRearch Laboratories, West Grove, PA, USA). The β-actin (1:5000, Santa Cruz Biotechnology) was used as an internal control. The bands were visualized with enhanced chemiluminescence reagents (West-Q pico Dura ECL solutions; GenDEPOT, Barker, TX, USA) under the LAS 4000 program (GE Healthcare, Pittsburgh, PA, USA).

### Immunocytochemistry

The endothelial cells onto the coated slide were fixed with 4% paraformaldehyde (PFA) for overnight at 4°C. After washing with PBS for three times, cells were blocked with 5% BSA for 1 h at room temperature. After washing with PBS, cells were reacted by the rabbit anti-PI3K (Santa Cruz Biotechnology), rabbit anti-pAkt (1:1000, Cell signaling), rabbit anti-Nrf-2 (1:1000, Santa Cruz Biotechnology), rabbit anti-HO-1 (1:1000, Abcam) and goat anti-Cox-2 (1:500, Santa Cruz Biotechnology) respectively at 4°C for 1 day, followed by FITC or Rhodamine-conjugated secondary antibody (1:1000, Jackson ImmunoResearch) at room temperature for 2 h. After washing with PBS, stained cells were mounted with Vectashield with DAPI (Vector Lab). Slides were observed under LSM 710 confocal laser scanning microscope (Carl Zeiss).

### Cell Viability

WST assay (EZ-CYTOX, Daeillab Service, Seoul, South Korea) was used for detecting cell viability of Neuro-2A cells. One day before performing the assay, the Neuro-2A cells were seeded in 96-well plates at a density 1 × 10^4^ cells per well with 100 μl of DMEM culture medium. After incubation with EC-CM for 24 h, WST (10 μl) in cultured media (100 μl) was added in each well. After 1 h 30 min reaction time at 37°C, the resultants were red by using VERSASA max microplate reader (Molecular Devices, Sunnyvale, CA, USA) at a wavelength 450 nm.

### MMP-9 Activity Assay

For the assessment of activity of MMP-9 in tissue, endothelial cell, and endothelia cell media, MMP-9 Biotrak Activity Assay system (Amersham Biosciences, Piscataway, NJ, USA) was used according to the manufacturer’s instructions. Tissue lysate, cell lysate, or cultured cell media was added into MMP-9 coated wells and incubated overnight for immunoreactivity at 4°C. To measure active MMP-9, detection enzyme was added and incubated at 37°C. The resultants were read by ELISA reader at 450 nm.

### Hoechst/PI Staining Assay

For detection of apoptosis in neuronal cells, Hoechst 33342/PI double staining was performed. Neuronal cells were incubated with Hoechst 33342 (10 μg/mL; ThermoFisher scientific) was or PI (Sigma-Aldrich) at 37°C for 10 min in the dark. After staining, cells were placed on a microscope slides and observed by LSM710 confocal microscope (Carl Zeiss). Hoechst 33342 is used for live cell staining of DNA and nuclei and PI is used for dead cell staining.

### Flow Cytometry

Neuro-2A cells were seeded in 100 mm dishes at a density 4 × 10^6^ cells and incubated with EC-CM for 24 h. Cells were washed with DPBS and collected. And then, cell suspensions were filtered through a cell strainer with a 40 μm nylon mesh. PI solution (10 μl) was added and incubated at room temperature for 10 min in the dark. Fluorescent cells (PI-positive) were determined immediately by using flow cytometer (LSR II, BD bioscience, San Hose, CA, USA). Flow cytometry data was analyzed using FlowJo version 10.

### Statistical Analysis

Data are expressed as the mean ± standard error of the mean (SEM). Statistical comparisons among the groups were assessed with a one way ANOVA followed by a Tukey *post hoc* test (Prism version 6.0 software). Statistical significance between groups was considered to be present at *p < 0.05, **p < 0.01, ***p < 0.001.

## Results

### Ischemic Injury Promotes Neuronal Cell Death at 24 h after Ischemia/Reperfusion

**FIGURE 1:**
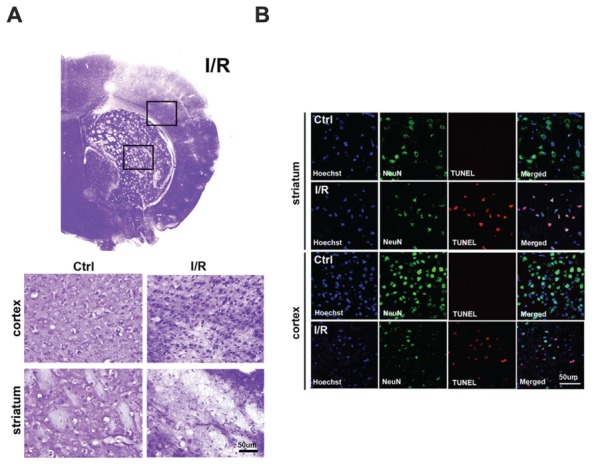
**Increased neuronal cell death in the brain after I/R. (A)** Morphological alterations were assessed by cresyl violet staining at 24 h after transient focal cerebral ischemia (tFCI). Round cell bodies were observed in the striatum and cortex of the control group, whereas thin and small cell bodies were noted in the striatum and cortex of the I/R group at 24 h after tFCI. **(B)** DNA fragmentation was labeled by TUNEL assays at 24 h after tFCI. NeuN-positive cells (green) and TUNEL-positive cells (red) were co-expressed in the striatum and cortex of the I/R group; however, TUNEL-positive cells were not easily found in these brain areas in the control group. Hoechst 33342 was used for counterstaining. TUNEL, terminal deoxynucleotidyl transferase dUTP nick end labeling.

We performed cresyl violet staining to assess the morphological alterations that occur in cells after ischemic injury (**Figure 1A**). In the control group, round and healthy cells were noted in the cerebral cortex and striatum, whereas in the I/R group, small and thin cell bodies were observed in the damaged cortex and striatum 24 h after ischemic injury. To examine whether ischemia induces neuronal cell death, we performed immunolabeling and TUNEL assays (**Figure 1B**). Compared to the control group, fewer neuronal nuclei NeuN-positive cells in the I/R group, were co-localized with TUNEL-positive cells in the striatum. Moreover, in the cortex, many TUNEL-positive cells were merged with NeuN-positive cells in the I/R group. However, TUNEL-positive cells (red) were not detected in the cortex and striatum of the control group. These results indicate that I/R injury promoted neuronal cell death in the lesioned brain areas at 24 h after I/R.

### Activated MMP-9 was Reversed by Blocking ASK1 Expression after Transient Focal Cerebral Ischemia

**FIGURE 2:**
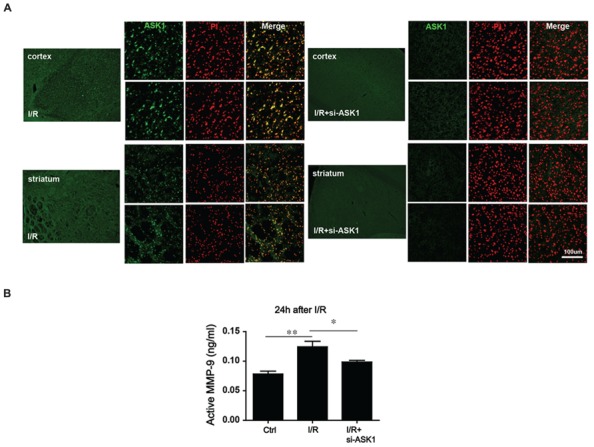
**Alteration in MMP-9 activity after ASK1 inhibition in the brain after I/R. (A)** Immnohistochemistry images show dense ASK1 expression in the striatum and cortex of mouse brain from the I/R group. After being treated with si-ASK1, the ASK1 level was efficiently diminished in the brains of the I/R+si-ASK group compared to the level in the I/R group. **(B)** MMP-9 activity was measured with an MMP-9 activity assay kit at 24 h after I/R *in vivo*. The elevated MMP-9 activity that was observed in the I/R group compared to that in the control group was alleviated by silencing ASK1 through genetic manipulations at 24 h after I/R (*n* = 6). [ASK1-siRNA sense, GCUCGUAAUUUAUACACUGtt; antisense, CAGUGUAUAAAUUACGAGCt; Bars represent mean ± SEM, *n* = 6. Active MMP-9 (ng/mL): control, 0.083 ± 0.004; I/R, 0.117 ± 0.008; I/R+si-ASK1, 0.101 ± 0.003. *p < 0.05, **p < 0.01, ***p < 0.001]. PI, propidium iodide, I/R, ischemia/reperfusion.

The previous study demonstrated that synthetic siRNA for ASK1 efficiently suppresses ASK1 and subsequently pASK1 after I/R (Kim et al., 2011). We used this method. To suppress the ASK1 level, siRNA (sense, GCUCGUAAUUUAUACACUGtt; antisense, CAGUGUAUAAAUUACGAGCtt) was injected through the intracerebroventricular route (Supplementary Figure S1). To confirm that ASK1 could be silenced efficiently by siRNA, we performed immunohistochemistry after tFCI. Our results showed that the increased ASK1 expression after ischemia was well-silenced by siRNA (**Figure 2A**). Next, to determine whether I/R and ASK1 could modulate MMP-9, we performed an MMP-9 activity assay at 24 h after I/R (**Figure 2B**). Our results showed that the level of active MMP-9 was significantly increased in the I/R group compared to the level in the control group. After silencing ASK1 with siRNA, the levels of active MMP-9 were efficiently attenuated in the brain tissue despite the I/R injury. Thus, ASK1 may contribute to MMP-9 activation at 24 h after I/R.

### MMP-9 Activity in Endothelial Cell-Conditioned Medium was Downregulated after the Inhibition of ASK1

**FIGURE 3:**
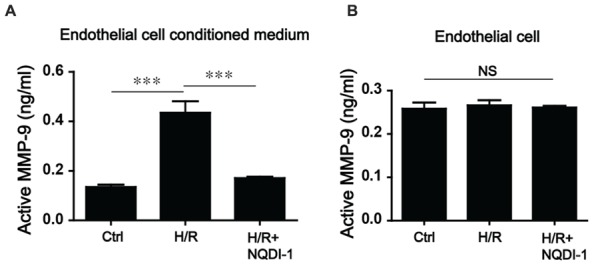
**Alterations of MMP-9 activity after inhibiting ASK1 in the EC-CM following H/R. (A)** In the EC-CM, MMP-9 activity was measured with an activity assay kit after H/R. After H/R, MMP-9 activity increased, but treatment with NQDI-1, an inhibitor of ASK1, restored the level of MMP-9 activity to that of the control group (*n* = 7). **(B)** In the cultured endothelial cells, no significant differences in MMP-9 activity were noted among the three groups (*n* = 3). [Bars represent mean ± SEM, Active MMP-9 (ng/mL) in EC-CM (*n* = 7): control, 0.135 ± 0.010; H/R, 0.435 ± 0.046; H/R+NQDI-1, 0.171 ± 0.199. Active MMP-9 (ng/mL) in endothelial cells (*n* = 3): control, 0.258 ± 0.014; H/R, 0.266 ± 0.012; H/R+NQDI-1, 0.261 ± 0.004. *p < 0.05, **p < 0.01, ***p < 0.001]. H/R, hypoxia/reperfusion.

The MMP-9 that is secreted from endothelial cells plays pivotal roles in BBB disruption, endothelial cell morphogenesis, and capillary formation (Dong et al., 2009; Dao Thi et al., 2012). Thus, we performed MMP-9 activity assay in cell media and cultured endothelial cells after 6 h (hypoxia)/24 h (reperfusion; **Figures 3A,B**). Our results showed that in EC-CM, the level of MMP-9 in the H/R group, was obviously increased compared to that in the control group. In the group treated with the ASK1 inhibitor NQDI-1, MMP-9 activity level was lower than that in the EC-CM of the H/R group (**Figure 3A**). However, in endothelial cells, the levels of active MMP-9 were not significantly different among the groups (**Figure 3B**).

### ASK1 Suppression Increased PI3K/Akt/Nrf-2/HO-1 Signaling Pathway and Decreased Cox-2 Signaling in Endothelial Cells

**FIGURE 4:**
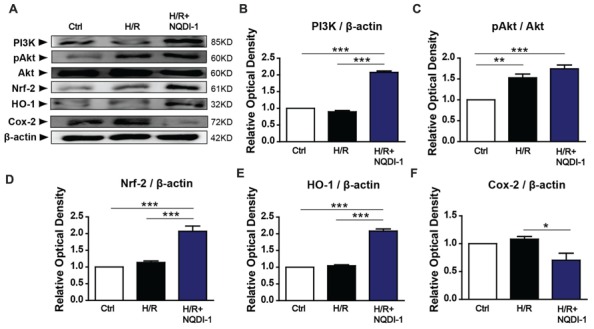
**Upregulated PI3K/Akt/Nrf-2/HO-1 signals and downregulated Cox-2 signals in endothelial cells after ASK1 inhibition and H/R. (A)** The amounts of PI3K, Akt, Nrf-2, HO-1, and Cox-2, as measured by Western blotting in the three groups. **(B)** Results of the semi-quantitative analysis showed that the PI3K level was higher in the H/R+NQDI-1 group than it was in the other groups (*n* = 3). **(C)** The protein level of pAkt was upregulated after H/R, but treatment with NQDI-1 significantly increased the pAkt level (*n* = 3). **(D,E)** Quantification of Nrf-2 and HO-1 revealed that the inhibition of ASK1 by NQDI-1 significantly increased the levels of these factors (*n* = 3). **(F)** The Cox-2 protein level in the H/R+NQDI-1 group was lower than that in the H/R group (*n* = 3-4). β-actin was used as an internal control. [Bars represent mean ± SEM, *n* = 3–4. Relative optical density (OD) of PI3K: H/R, 0. 90 ± 0.04; H/R+NQDI-1, 2.08 ± 0.04. OD of pAkt: H/R, 1.53 ± 0.09; H/R+NQDI-1, 1.74 ± 0.09. OD of Nrf-2: H/R, 1.14 ± 0.05; H/R+NQDI-1, 2.07 ± 0.16. OD of HO-1: H/R, 1.05 ± 0.03; H/R+NQDI-1, 2.08 ± 0.06. OD of Cox-2: H/R, 1.08 ± 0.05; H/R+NQDI-1, 0.70 ± 0.13. *p < 0.05, **p < 0.01, ***p < 0.001]. H/R, hypoxia/reperfusion.

**FIGURE 5:**
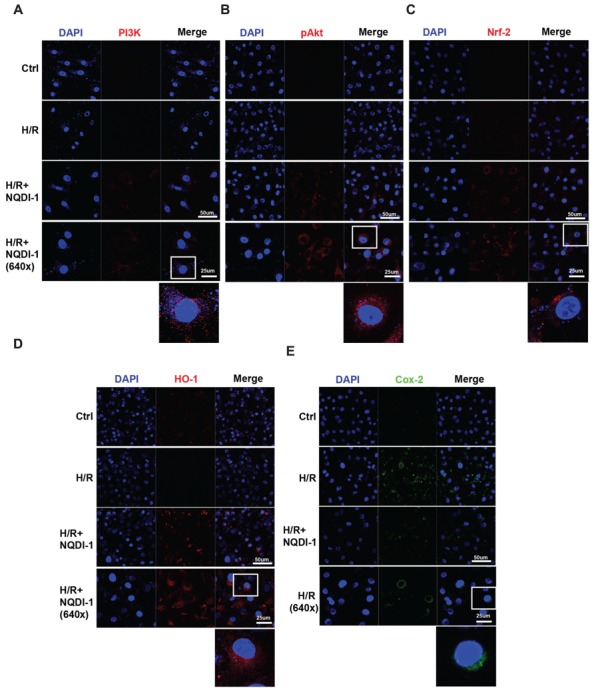
**Elevated PI3K/Akt/Nrf-2/HO-1 expressions and reduced Cox-2 expression in endothelial cells after ASK1 inhibition and H/R.** The levels of PI3K/Akt/Nrf-2/HO-1 and Cox-2 expression were evaluated by immunocytochemistry after H/R. **(A)** Representative image showing that following H/R, PI3K expression (red) was markedly observed after inhibiting ASK1 with NQDI-1 compared to the expression levels in the control and H/R groups. **(B–D)** Confocal image demonstrating that ASK1 inhibition increased Akt, Nrf-2, and HO-1 expression, respectively. **(E)** Blocking ASK1 reduced Cox-2 expression (green) in endothelial cells after H/R. DAPI was used as a counterstain. H/R, hypoxia/reperfusion.

To elucidate the mechanisms underlying the protective effects of ASK1 inhibition against MMP-9 in endothelial cells during H/R, we performed Western blot analysis (**Figure 4**) and immunocytochemistry (**Figure 5**). A previous study demonstrated that exposure to oxidative stress induces PI3K/Akt signaling, which activates pro-survival reactions after stimuli (Wang et al., 2007). Based on this previous study, we assessed the PI3K protein levels after oxidative stress in endothelial cells. However, the PI3K protein level of these cells was not altered after exposure to H/R. Interestingly, the PI3K level in NQDI-1-treated cells was significantly increased at 24 h after H/R (**Figures 4A,B**). Using Western blots, we also measured the level of Akt protein, which is downstream of PI3K. Although the quantitative graph showed that the level of phosphorylated-Akt (pAkt) in the H/R group was upregulated, compared to the level in the control group, after treatment with NQDI-1, the pAkt level increased further despite the H/R injury (**Figures 4A,C**). Next, we hypothesized that PI3K/Akt as upstream signaling molecules signals would modulate the Nrf-2/HO-1 pathway during hypoxia injury (Martin et al., 2004; Wu et al., 2013; Meng et al., 2014; Xu X.H. et al., 2015). The results of our Western blot analysis revealed that the, Nrf-2 protein level was also significantly upregulated, compared to the level in the H/R group after blocking ASK1 with NQDI-1 (**Figures 4A,D**). The HO-1 protein levels in the H/R group were not increased compared to the levels in the control group; however, blocking ASK1 increased the HO-1 levels regardless of the H/R injury (**Figures 4A,E**). It has been recognized that Cox-2 is an important mediator of ischemic injury development in the brain (Nogawa et al., 1997). Our data indicated that following H/R injury, the Cox-2 protein levels were reduced after treatment with NQDI-1 (**Figures 4A,F**). Next, we performed immunocytochemistry. Our data demonstrated that PI3K-positive cells (red) showed markedly decreased immunoreactivity in the H/R group. On the other hand, PI3K expression in the H/R+NQDI-1 group was enhanced, compared to the expression in the control and H/R groups (**Figure 5A**). Additionally, pAkt (red) was highly expressed in a cytosol-like pattern in the H/R+NQDI-1 group, compared to in the H/R group (**Figure 5B**). We also observed lower Nrf-2 immunoreactivity in the H/R group. After blocking the ASK1 signal, the expression of Nrf-2 in the H/R+NQDI-1 group was increased compared to that in the H/R group (**Figure 5C**). No HO-1 expression was observed in the H/R group, but intense HO-1 expression was noted in the H/R+NQDI-1 group (**Figure 5D**). In contrast, Cox-2 expression was upregulated in H/R-injured endothelial cells, but was downregulated after ASK1 inhibition despite the H/R injury (**Figure 5E**). Collectively, our data suggest that ASK1 inhibition reduces Cox-2 signals but increases PI3K/Akt/Nrf-2/HO-1 signaling after H/R injury in endothelial cells.

### Apoptotic Molecules Were Attenuated after Incubation with ASK1 Inhibitor-Treated EC-CM

**FIGURE 6:**
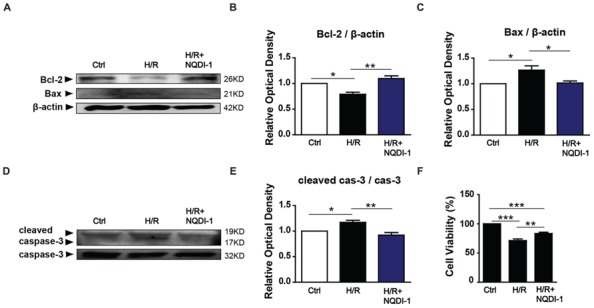
**Reduction of apoptotic molecules following the inhibition of ASK1. (A)** The levels of anti-apoptotic (Bcl-2) and pro-apoptotic (Bax) proteins were detected with Western blot analyses. **(B)** The relative optical density indicated that neuronal Bcl-2 expression was reduced after incubating the cells with EC-CM from the H/R group. After incubation with NQDI-1-treated EC-CM, the Bcl-2 level was increased (*n* = 3). **(C)** The increased Bax level that was observed after incubating the cells with H/R-injured EC-CM was reduced after NQDI-1 treatment (*n* = 3-4). **(D)** The level of apoptotic (caspase-3) protein was measured by Western blot analyses. **(E)** The graph showed the cleaved caspase-3 was increased in H/R-injured EC-CM, whereas ASK1-inhibited group exhibited lower cleaved caspase-3 level in neuronal cells (*n* = 3). **(F)** Cell viability was detected with viability assays. The quantitative analyses show that neuronal cells were significantly dead after incubation with H/R-injured EC-CM; however, the cell viability was increased by treatment with ASK1 inhibitor (*n* = 7). β-actin was used as an internal control. [Bars represent mean ± SEM, *n* = 3–4. Relative optical density (OD) of Bcl-2: H/R, 0. 79 ± 0.04; H/R+NQDI-1, 1.10 ± 0.05. OD of Bax: H/R, 1.27 ± 0.08; H/R+NQDI-1, 1.01 ± 0.04. OD of cleaved caspase-3: H/R, 1.17 ± 0.04; H/R+NQDI-1, 0.92 ± 0.05. *p < 0.05, **p < 0.01, ***p < 0.001]. H/R, hypoxia/reperfusion; EC-CM, endothelial cell-conditioned medium.

To elucidate whether ASK1 and MMP-9 modulate neuronal cell fate, we treated neuronal cells with EC-CM and performed Western blot analyses and cell viability assays. We measured the protein levels of B-cell lymphoma 2 (Bcl-2) an anti-apoptotic marker, and Bcl-2-associated X (Bax), a pro-apoptotic marker, and caspase-3, an apoptotic molecule, in the neuronal cell culture (**Figure 6**). The quantitative analysis demonstrated that the Bcl-2 level was decreased in neuronal cells incubated with H/R-injured EC-CM. However, neuronal cells incubated with H/R+NQDI-1-treated EC-CM showed efficiently increased Bcl-2 levels (**Figure 6B**). In contrast to the results for Bcl-2, the Bax expression level was obviously increased in neuronal cells exposed to H/R-injured EC-CM. After attenuating of ASK1 in the EC-CM, the Bax protein level was downregulated in neuronal cells (**Figure 6C**). Cleaved caspase-3 level in neuronal cells was elevated in neuronal cells incubated with H/R-injured EC-CM, this level was reversed after H/R+NQDI-1-treated EC-CM (**Figure 6E**). To investigate the neuronal cell viability, viability assays were conducted at 24 h after EC-CM incubation (**Figure 6F**). After normalizing the cell viability with the control values, the cell viability (%) of neuronal cells incubated with the H/R group was 71.32%; however, the cell viability increased to 83.42% after incubation with ASK1 inhibitor-treated EC-CM. These results indicated that apoptotic factors were downregulated and anti-apoptotic factor was upregulated in neuronal cells after inhibition of ASK1.

### Neuronal Cell Death was Decreased after Incubation with ASK1 Inhibitor-Treated EC-CM

**FIGURE 7:**
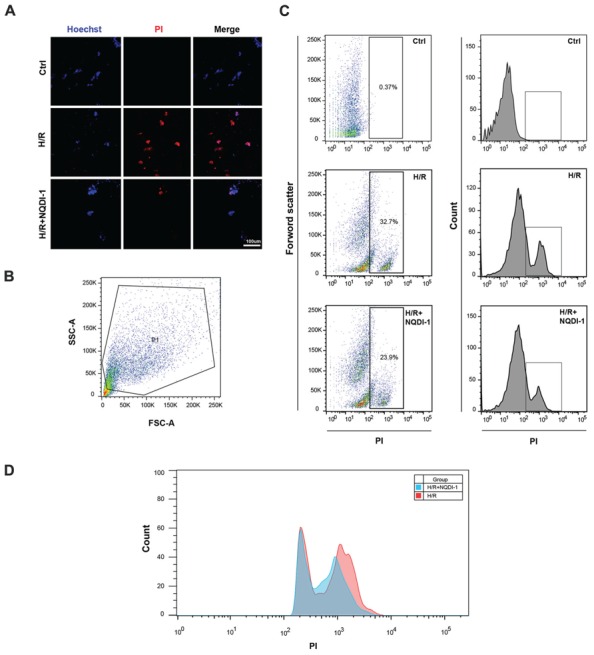
**Decrease of neuronal cell death following the inhibition of ASK1. (A)** Using Hoechst33342/PI staining, dead cell (PI-positive, red) was observed by confocal microscope. PI-positive cells rarely detected in the H/R+NQDI-1 group compared to that in the H/R group. **(B)** Representative flow cytogram showed the gating for PI-positive cell analysis. **(C,D)** Dot plot and histogram showed that many PI-positive cells was sorted in the H/R group, however, relatively lower PI-positive cells were detected in the H/R+NQDI-1 group. PI, propidium iodide; H/R, hypoxia/reperfusion.

To confirm that the apoptosis-related molecules regulated by ASK1 lead to neuronal cell death, we performed Hoechst/PI dual staining and FACS analysis (**Figure 7**). Confocal images showed that PI-positive cells (red) were found easily in neuronal cells which incubated with EC-CM after H/R, however, neuronal cells with ASK1 inhibited EC-CM showed lower PI-positive cells and in the control group, we cannot observe PI-positive cells (**Figure 7A**). Single cell suspensions were labeled with PI and analyzed by flow cytometry after incubation with EC-CM. A primary gating was based on forward and side light scatter respectively and cell debris were excluded (**Figure 7B**). Sorted cells were measured by PI fluorescence laser. The dot plot showed that neuronal cells in the H/R group showed a much higher amount of PI-positive cells (cell percentage; 32.7%) compared to those in the H/R+NQDI-1 group (cell percentage; 23.9%; **Figure 7C**). In addition, merged histogram exhibited increased PI-positive cell counts in the H/R group, compared to those in the H/R+NQDI-1 group (**Figure 7D**). In the ischemic brain tissue, after silencing ASK1, a marked reduction in apoptotic cell death was detected by using a TUNEL assay *in vivo* (Supplementary Figure S2). These data suggest that neuronal cell death was ameliorated by inhibiting ASK1.

## Discussion

**FIGURE 8:**
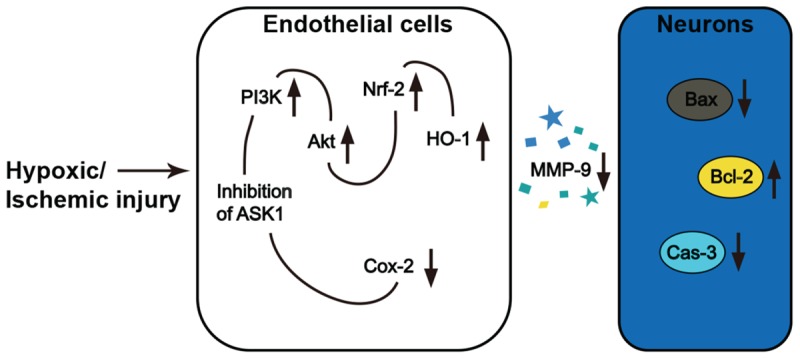
**Inhibition of ASK1 reduced MMP-9 activity and subsequent neuronal cell death after ischemic or hypoxic injury**.

In the pathology of cerebral ischemia, oxidative stress is one of the major causes of brain damage and neuronal cell death. Many therapeutic approaches for addressing oxidative stress-induced BBB disruption and neuronal cell death have been developed. However, no curative strategies currently exist. In the present study, neuronal cell death occurred in the ipsilateral hemispheres of mice brains that were exposed to ischemic insults, and this finding was in accordance with the results of several previous studies (Kim et al., 2011; Cheon et al., 2013). Within the ischemic lesion, the level of MMP-9, which is known to be secreted by brain endothelial cells, was elevated at 24 h after I/R. Similarly, we detected activated MMP-9 after H/R injury in endothelial cell media. However, silencing ASK1, an early responder in the intracellular cascade that is related to oxidative stress, ameliorated MMP-9 activation in both the ischemic brain and endothelial cell media. To elucidate the mechanisms underlying the ASK1-induced MMP-9 activation, we genetically and chemically inhibited ASK1. Our results in cultured brain endothelial cells demonstrated that ASK1 inhibition leads to the upregulation of PI3K/Akt/Nrf-2/HO-1 and the downregulation of Cox-2 signals in intracellular cascades (**Figure 8**). We also found that neuronal cells incubated with H/R treated EC-CM, in which high levels of active MMP-9 were detected, showed increased levels of the pro-apoptotic molecule Bax and caspase-3, significantly lower cell viability, and higher PI-positive cells than the control group. However, these results were reversed following the blockade of ASK1 and the subsequent suppression of active MMP-9. After incubating cultured neuronal cells with NQDI-1 treated EC-CM, higher expression of the anti-apoptotic molecule Bcl-2 was noted, the cell viability was higher, and PI-positive cells were lower than those of the H/R group. In addition, DNA fragmentation, a characteristic of apoptosis, was attenuated in various brain regions including the cerebral cortex and striatum after ischemic stroke. Together, our data demonstrated that inhibiting ASK1 suppressed the secretion of active MMP-9 from injured endothelial cells and contributed to the observed reduction in neuronal cell death after ischemic insult.

Following cerebral ischemia, neuronal cell death occurs in the affected areas. MMP-9 expression is the main factor contributing to the progression of neuronal apoptosis and the subsequent brain infarct (Romanic et al., 1998; Dong et al., 2009). Our data show that blocking ASK1 reduces the secretion of MMP-9 from in endothelial cells. These findings indicate that ASK1 may contribute to the signaling pathway that is associated with MMP-9 activation. The molecular mechanisms underlying the induction of MMP-9 by ASK1 in endothelial cells are currently unknown, however, previous studies demonstrated that MMP-9 activation may be associated with Cox-2 signals (Dong et al., 2009). Indeed, ours study reveal that ASK1 inhibition induced decreases in Cox-2, thus suppressing MMP-9. Therefore, MMP-9 activation may be inhibited via the amelioration of the ASK1-Cox-2 pathway. Although relationship between ASK1 and Cox-2 is little established in ischemic model, in macrophage cell line, ASK1 is associated to Cox-2 expression following peptidoglycan stimulation (Hsu et al., 2010). In addition, several studies reported that Cox-2 regulates vascular endothelial cell function and external stimuli, such as lipopolysaccharide, Cox-2 expression increases in brain microvascular endothelial cells (Williams et al., 1999; Ejima et al., 2003). Cox-2 is also an important contributor to the progression of brain injury after cerebral ischemia, as studies have shown that the pharmacological or genetic silencing of Cox-2 results in decreased brain damage, while the overexpression of Cox-2 increases the brain’s vulnerability to ischemic injury (Nogawa et al., 1997; Dore et al., 2003; Sasaki et al., 2004; Kunz et al., 2007). Furthermore, a recent study demonstrated that inhibitors of Cox-2 decrease the expression of active-MMP-9 in neuronal and endothelial cells and prevent edema formation in the brain after ischemia (Dong et al., 2009).

Heme oxygenase-1 is recognized as a promising therapeutic target in oxidative stress induced pathological conditions (Aztatzi-Santillan et al., 2010). HO-1 is a cytoprotective phase II enzyme that exerts anti-apoptotic and anti-oxidant effects on the vascular system and controls the function of the BBB via its interactions with proteases (Lin et al., 2007; Chung et al., 2008). Here, ASK1 inhibition induced HO-1 expression and suppressed MMP-9 activity in endothelial cell cultures. A previous study showed that Nrf-2 mediates phase II enzyme activity as an upstream molecule of HO-1 and increases HO-1 expression under conditions of oxidative stress (Xu X. et al., 2015). The inhibition of Nrf-2 by brusatol resulted in damaged Nrf-2-mediated defense activity (Ren et al., 2011; Xu X. et al., 2015). Several studies demonstrated that the PI3K/Akt signaling pathway contributes to cellular defense and cell survival, as well as modulates Nrf-2 as an upstream signal (Martin et al., 2004; Ren et al., 2011; Xu X. et al., 2015). Blocking the PI3K/Akt signaling pathway with LY294002 reduces HO-1 expression in lipopolysaccharide-induced macrophages (Xu X. et al., 2015). Additionally, in an experimental mimic model of ischemic stroke, the upregulation of PI3K/Akt meditated Nrf-2 signaling pathway causes further HO-1 activation and neuroprotective effects (Martin et al., 2004; Wu et al., 2013; Meng et al., 2014; Xu X.H. et al., 2015). It is also well-known that Akt is a negative regulator of ASK1 activity under ischemic conditions (Wang et al., 2007) and that Akt attenuates ASK1 activity by directly phosphorylating at Ser-83 (Wang et al., 2007). Therefore, through the phosphorylation of Ser-83 in an ASK1-dependent manner, the ASK1/c-Jun N-terminal kinase pathway was attenuated via PI3K/Akt activation (Wang et al., 2007). In contrast, a previous study demonstrated that PI3K/Akt signaling was inhibited by ASK1 (Subramanyam et al., 2010). Although it is not clear whether Akt or ASK1 is controlled as an upstream molecule, we found that ASK1 is closely related to Akt mechanistically. Our results imply that the ASK1 blockade may have enhanced the defense system by upregulating PI3K/Akt/Nrf-2 and HO-1 signals and downregulating Cox-2 signals, which is likely to reduce the secretion of MMP-9. However, our findings showed only expression changes in endothelial cell, therefore, it is difficult that MMP-9 suppression was directly induced by PI3K/Akt/Nrf-2/HO-1 and Cox-2. This investigation is our further study.

Although, previous study proved that siRNA for ASK1 has an efficient capability for reducing ASK1 level and ASK1 silencing contributes to cell survival (Kim et al., 2011). The protective action mechanism by blocking ASK1 in neuronal cells has not been fully elucidated. Important point from our results is that endothelial ASK1 can modulate neuronal cell signalings. It is likely that active MMP-9 secretion from endothelial cells contribute to ASK1 mechanism. The early phase of MMP-9 inhibition by siRNA blocks MMP-9 secretion from endothelial cells, leading to a decreased the infarct volume (Hu et al., 2009). On the contrary, MMP-9 expression exacerbates ischemic brain injury (Romanic et al., 1998). Similar to previous studies, in our results, the inhibition of MMP-9 activation by ASK1 blocking showed an effect on neuronal cell fate in response to ischemic injury. After incubation with endothelial cell media after H/R, which contained high level of active MMP-9, the levels of the anti-apoptotic Bcl-2 were reduced while the levels of the pro-apoptotic Bax and apoptotic caspase-3 were increased in neurons. Higher amounts of apoptotic molecules could promote neuronal apoptosis. After ASK1 blocking, Bcl-2 was upregulated and Bax and caspase-3 was downregulated, and these changes might be modulated by ASK1 inhibition and MMP-9 suppression. Subsequent neuronal cell death was reduced. Furthermore, in ischemic brain, easily observed apoptotic cell death was reversed by the silence of ASK1.

Collectively, our findings provide that inhibition of ASK1 might be a novel approach that can protect against vascular damage and neuronal cell death after cerebral ischemia. Since the brain injury that occurs after cerebral ischemia is a result of neuronal cell death with contributions both ASK1 and MMP-9 activation. Inhibition of ASK1 could be an efficient strategy for attenuating MMP-9 activation and preventing neuronal cell death after ischemic stroke.

## Author Contributions

B-NK designed this study, interpreted all data and wrote manuscript. JL participated in design of animal study and interpreted some data. SC participated in the collection of data, data analysis, and manuscript writing. KC, SK, and EK participated in the data interpretation. All authors read and approved final manuscript.

## Conflict of Interest Statement

The authors declare that the research was conducted in the absence of any commercial or financial relationships that could be construed as a potential conflict of interest.

## Abbreviations

ASK1: apoptosis signal-regulating kinase 1
BBB: blood–brain barrier
CNS: central nervous system
Cox-2: cyclooxygenase-2
EC-CM: endothelial cell-conditioned medium
HO-1: heme oxygenase-1
H/R: hypoxia/reperfusion
MMP: matrix metalloproteinase
Nrf-2: nuclear factor erythroid 2 [NF-E2]-related factor 2
OGD: oxygen–glucose deprivation
PI3K: phosphatidylinositol 3-kinase
ROS: reactive oxygen species.
