# Supplementary material for: Blockade of Apoptosis Signal-Regulating Kinase 1 Attenuates Matrix Metalloproteinase 9 Activity in Brain Endothelial Cells and the Subsequent Apoptosis in Neurons after Ischemic Injury
Source: Front Cell Neurosci. 2016 Sep 2;10:213. doi: 10.3389/fncel.2016.00213 (PMC5009117; doi:10.3389/fncel.2016.00213)
Supplement: Supplementary file 1 [file Data_Sheet_1.DOCX]

**Supplementary data**

**
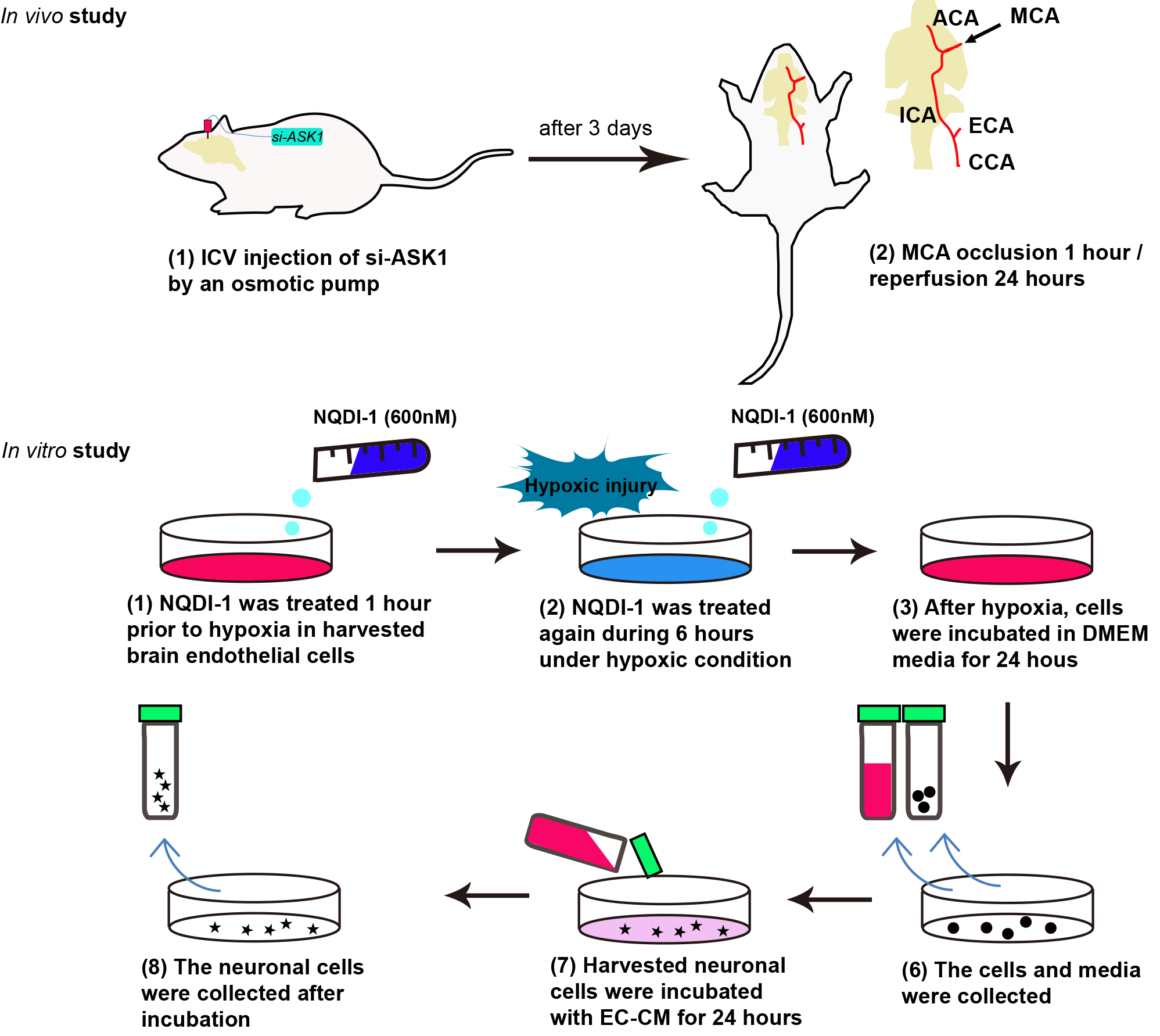
**

**Figure S1. Experimental procedures.** An osmotic pump containing siRNA for ASK1 was placed on the back of a mouse and the siRNA was injected into the lateral ventricle for 3 days. Cerebral ischemia (I) was induced by occluding the MCA for 1 h and then permitting reperfusion (R) for 24 h. The mice were divided into 3 groups, as follows:1) control group, 2) I/R group, and 3) siRNA for ASK1-treated I/R (I/R+si-ASK1) group. For the i*n vitro* study, mouse endothelial cells (bEnd3 cell line) were subjected to hypoxia injury for 6 h. An inhibitor of ASK1, NQDI-1, was applied for 1 h prior to the hypoxia exposure and for 6 h during hypoxia. After the injury, cells were returned to the normoxic condition for 24 h. The endothelial cells and endothelial cell culture conditioned media (EC-CM) were collected. Neuronal cells (Neuro2A) were cultured with collected EC-CM for 24 h.


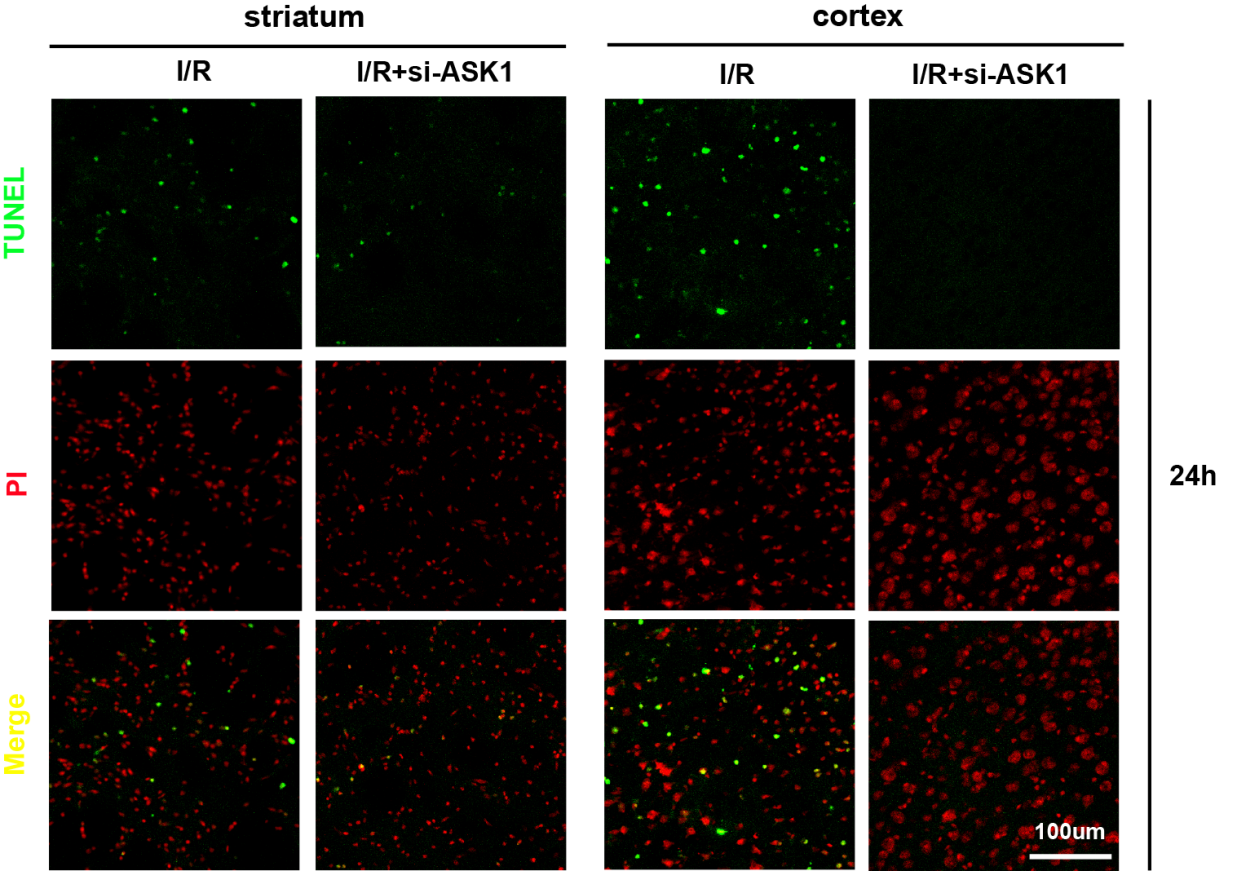


**Figure S2. Reduction of apoptotic cell death after ASK1 silencing in cerebral ischemia.** Changes in apoptotic cell death after silencing of ASK1 were observed via TUNEL assays. Overexpressed TUNEL immunoreactivity (green) was detected in the ischemic striatum and cortex at 24 h after I/R. After silencing ASK1, TUNEL-positive cells were rarely observed in the ischemic brain. PI, propidium iodide, I/R, ischemia/reperfusion.
